# The function of CD36 in *Mycobacterium tuberculosis* infection

**DOI:** 10.3389/fimmu.2024.1413947

**Published:** 2024-05-31

**Authors:** Jianjun Wang, Hui Cao, Hongwei Yang, Nan Wang, Yiwei Weng, Hao Luo

**Affiliations:** ^1^ Department of Clinical Laboratory, Kunshan Hospital Affiliated to Jiangsu University, Suzhou, China; ^2^ Department of Food and Nutrition Safety, Jiangsu Provincial Center for Disease Control and Prevention, Nanjing, Jiangsu, China; ^3^ Department of Clinical Laboratory, Suzhou BOE Hospital, Suzhou, Jiangsu, China; ^4^ Department of Clinical Laboratory, The Fourth People’s Hospital of Kunshan, Suzhou, Jiangsu, China; ^5^ Department of Clinical Laboratory, The Second People's Hospital of Kunshan, Suzhou, China

**Keywords:** CD36, *Mycobacterium tuberculosis*, inflammatory response, immune response, lipid metabolism, biomarker

## Abstract

CD36 is a scavenger receptor that has been reported to function as a signaling receptor that responds to pathogen-associated molecular patterns (PAMPs) and damage-associated molecular patterns (DAMPs) and could integrate metabolic pathways and cell signaling through its dual functions. Thereby influencing activation to regulate the immune response and immune cell differentiation. Recent studies have revealed that CD36 plays critical roles in the process of lipid metabolism, inflammatory response and immune process caused by *Mycobacterium tuberculosis* infection. This review will comprehensively investigate CD36’s functions in lipid uptake and processing, inflammatory response, immune response and therapeutic targets and biomarkers in the infection process of *M. tuberculosis*. The study also raised outstanding issues in this field to designate future directions.

## 1 Introduction

*Mycobacterium tuberculosis* (*M. tuberculosis*) as an intracellular pathogen is a threat to global public health and infects approximately a third of the world’s population and it causes approximately 2 million deaths every year around the world (1, 2). The pathogenic mycobacteria has the potential to adapt to the hostile intracellular environment of macrophages and make it latent to infect cells in the surrounding environment (3–5). *M. tuberculosis* involves host trafficking pathways by regulating maturation pathways such as phagosomal/endosomal to generate a protected niche for itself, the mycobacterial phagosome (6–8). Furthermore, *M. Tuberculosis* regulates the specific metabolic pathways to subvert the host signaling response, thus regulating the host immune response and enabling access to nutrients, which ultimately favor infection establishment (9, 10). The macrophages are critical immune cells in *M. tuberculosis* infection and the foam-like macrophages have been found in the granulomatous structures in both *M. tuberculosis-*infected human disease and animal models (11–13). The foam of macrophages in an *M. tuberculosis-*infected environment reflected the disruption of intracellular lipid regulatory mechanisms through the observation of infected pathological conditions. Numerous studies have shown that during *M. tuberculosis* infection, newly generated lipid bodies are often architecturally unique cytoplasmic organelles involved in the manufacture of lipid mediators with immunomodulatory effects (14, 15). In addition, the induction and targeting of liposomes by *M. tuberculosis* may provide an escape mechanism during infection due to the downregulation of the immune response and/or nutrient uptake in macrophages, promoting the survival and replication in host cells (16, 17). Interestingly, the scavenger receptor CD36 is closely related to the formation of foam-like macrophages and their lipid metabolism during the infection of *M. tuberculosis*.

Cell surface glycoprotein CD36 is a scavenger receptor present in various cells and functions in various roles in lipid metabolism, mediating lipid acquisition, immune recognition, inflammation, molecular adhesion and apoptosis (18–20). It has associations with angiogenesis, atherothrombotic disease, metabolic disorders, diabetes, obesity and other conditions. By sensing a range of microbial components and endogenous ligands, it may operate as a pattern recognition receptor mediating innate immune or inflammatory responses to a variety of pathogens (21, 22). Researches have shown that CD36 may also function as a co-receptor with the Toll-like receptor (TLR) 2/6 complex, which is involved in innate sensing, lipoteichoic acid binding, and *Staphylococcus aureus* phagocytic clearance (23, 24). Additionally, a growing body of research has revealed that patients with active tuberculosis (ATB) exhibit reversible changes in CD36 on their peripheral macrophages/monocytes (25). All in all, the significant role of CD36 is to recognize the ligands from the pathogen or host itself to induce the appropriate immune responses to protect the host itself and eliminate the pathogens.

However, the tangible molecular mechanisms and signaling pathways which regulate lipid body biogenesis during *M. tuberculosis* infection and the contribution it has to tuberculosis’ pathophysiology are not clear. This study will delve into and review the functions and specific signaling mechanisms of CD36 in *M. tuberculosis*, providing comprehensive materials for the study of CD36 in *M. tuberculosis* infection

## 2 The biological functions of CD36 molecular

**Figure 1:**
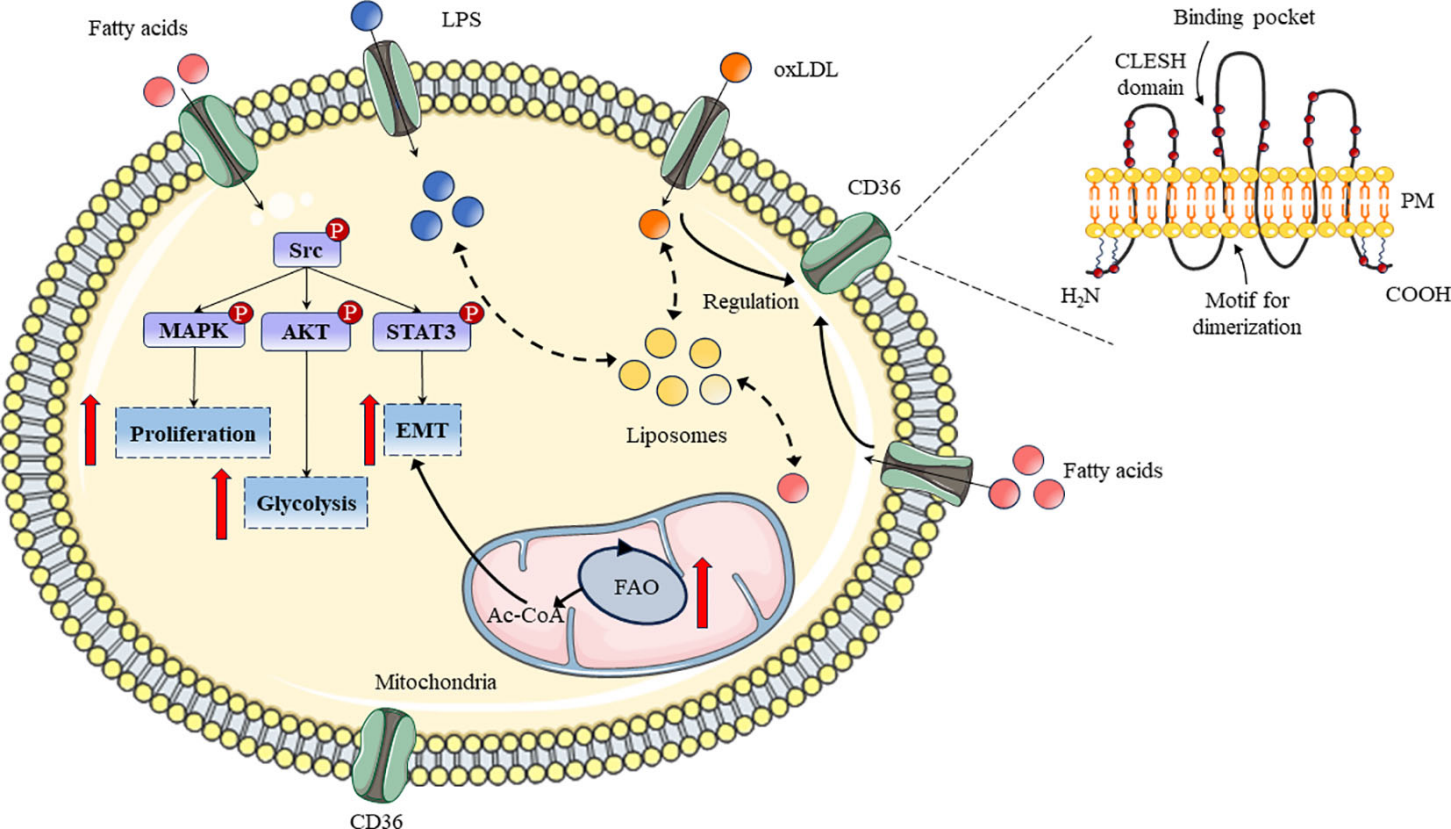
Schematic diagram of CD36 structure and its roles in lipid metabolism process. The structure and related functional regions of CD36 in bilayer lipids were displayed. Meanwhile, the pathways, related signaling pathways, and functions of CD36 in lipid metabolism have been demonstrated.

As a 55 kDa heavily glycosylated protein, the membrane glycoprotein CD36 has two distinct structural parts including extracellular ectodomain and cytosolic domain respectively (26). For a specific collection of adaptor proteins, the extracellular ectodomain serves as a docking location where the signal is relayed for functional outcomes during the signaling pathway initiated at the cell surface via CD36-ligand contact (27). Transmembrane sections link the protein’s ectodomain and cytosolic (C- or N-terminal) portions (**Figure 1**). Following ligand-receptor contact, CD36 is internalized with or without ligand and the reaction is amplified by the internalized CD36-ligand complex (28).

Scavenger receptors have the special ability to collect cellular waste or dead/senescent cells which are then disposed of in the target spleen (29). Moreover, CD36’s potential is dependent on its ability to bind with ligands found in cells or cellular debris, as well as how well this interaction is eliminated (30). The physiochemical aspects of topology, charge and other characteristics of the CD36 ectodomain enable it to recognize a wide variety of ligands, including phosphatidylserine, oxidized low-density lipoprotein (oxiLDL), lipopolysaccharide (LPS), long-chain fatty acids (LCFAs) and so on (31–34). CD36 surface chemistry and ectodomain topology could also facilitate the ligands binding that have diversified unknown origins.

CD36 could be expressed on the surface of various cell types including monocytes, macrophages, cancer cells, endothelial cells and platelets (35). Circulating monocytes in the blood are transmigrated into the arterial intima and differentiated into macrophages, but CD36 expression in macrophages is regulated by various factors (36). In addition, multitudinous chemical compounds such as curcumin, astaxanthin, quercitrin and kaempferol could regulate CD36 expression through erythroid-related factor 2 (Nrf2) and peroxisome proliferator-activated receptor-gamma (PPAR-γ) signaling pathways in macrophages (37–39). In cancer cells, CD36 could also regulate cell proliferation, glycolysis, and Epithelial-Mesenchymal Transition (EMT) through the MAPK, AKT, and STAT3 signaling pathways to determine the physiological and pathological processes of cells (**Figure 1**) (40–42). In addition, to reduce the parasite burden, a combination of CD36 ligands generates pro-inflammatory cytokines; however, this improper cytokine production leads to pathogenic alterations (43). Undoubtedly, CD36 and its downstream signaling may offer useful resources for developing appropriate adjuvant treatments that aid in the recovery from pathology linked to the disease (44). Furthermore, specific downstream signaling can be triggered by a suitable chemical substance acting as a ligand which will generate an appropriate immune response to eradicate the infection (45).

Macrophages have high expression of CD36 which is intimately associated with their multitude of functions. Because monocyte/macrophage CD36 may bind to and increase oxLDL endocytosis as well as take part in the generation of foam cells, it plays a crucial role in the development of atherosclerotic lesions (46). Macrophages could bind and ingest ox-LDL with the assistance of CD36, and the internalized ox-LDL activates PPAR-γ to upregulate CD36 expression, which facilitates the uptake of ox-LDL further (35, 47, 48). Foam cells and the accumulation of esterified cholesterol in macrophages are caused by elevated ox-LDL (49). Additionally, the uptake of ox-LDL by CD36 sets off cytokine production and immune cell recruitment to the artery intima, which leads to arterial narrowing and the advancement of atherosclerotic vascular disease (50). A significant amount of foam cells are formed *in vitro* and *in vivo* due to CD36 and blocking CD36 expression or downstream signaling prevents animals from absorbing ox-LDL which limits the progression of experimental atherosclerosis in mice. A link between carotid atherosclerosis and the plasma levels of soluble CD36 (sCD36) was discovered by Handberg et al. (51).

The function of CD36 is to identify ligands from the pathogen or the host, at which point it triggers the right innate immune response that kills the infection and produces inflammatory cytokines (52). In the immune process initiated by pathogenic infections, the recognition of TLR2 agonists is affected by some accessory receptors, such as CD36, CD11b/CD18 and CD14 (53, 54). Moreover, the identification of co-receptors that promote TLR functions reveals that TLRs combine with other cell surface molecules to link PAMP recognition to the initiation of inflammatory responses (55). Hoebe et al. demonstrated that TLR2/TLR6 heterodimers require CD36 and the heterotypic binding of TLR2/6 to CD36 is not pre-formed, but ligand-induced, revealing the heterotypic association of TLR2 (56, 57). Moreover, deposition of the altered self-components oxLDL and amyloid-beta induce inflammatory response through CD36-triggered the heterodimer activation of TLR4-TLR6 in Alzheimer’s disease and atherosclerosis, indicating a novel model of TLR heterodimerization dominated by co-receptor signaling factors (58). These studies indicated that the function of CD36 cannot be achieved without the coordination of the TLR receptor family.

Recent research on CD36’s function in infectious disorders has shown that it plays a pathological role in infections with viruses, tuberculosis, pneumonia and *Staphylococcus aureus*. In influenza virus-mediated pneumonia, the expression of CD36 was downregulated and since the host is more susceptible to infection when CD36 is absent, CD36-mediated phagocytosis is required to eliminate germs (59). Moreover, the residues of the cytoplasmic domain (Y463 and C464) were critical and involved in TLR2/6 signaling (60). Meanwhile, CD36-deficient macrophages failed to eliminate the bacteria and decreased the secretion of TNF main components revealed that the cytoplasmic domain of the CD36 receptor is required for the phagocytosis (60). Additional findings revealed that CD36 expression may be associated with HIV infections, influenza and hepatitis virus (61, 62). HBV replication is facilitated by the store-operated Ca^2+^ channel, which is mediated by the Src-kinase-mediated signaling pathway and is mediated by CD36 (63). The Nef protein of HIV downregulated CD36 which impacted the infected cells’ ability to be phagocytosed and added to the host’s pathology by pointing to the TNF-α and co-interactions of the infected cells (64).

Currently, an increasing number of studies have shown abnormal expression of CD36 in *Mycobacterium* infection, but the function of CD36 in *Mycobacterium* infection is still insufficient and ambiguous. Fink et al. discovered via gene sequencing that CD36 is down-regulated in zebrafish infected with *M. marinum*; conversely, the knockdown of CD36 defective zebrafish larvae resulted in a higher bacterial burden during such infection (65). Bazzi et al. reported downregulating CD36 expression, reducing CCL2 spontaneous release and increasing CCL5, CXCL8/IL-8, IL-6 and TNF-α spontaneous secretion in *M. obuense* infected macrophages (66). Park et al. found that macrophages expressed CD14, CD36 and TLR2, and function the most active responses against *M. avium subsp. paratuberculosis* (MAP) infection, which includes the expression of proinflammatory cytokines and chemokines such as CCL4, CCL3, IL-1b, IL-8, and CCL20 (67). CD36 plays an indispensable role in macrophage resistance against *Mycobacterium* infection, and the abnormality of CD36 is closely related to the inflammatory response of cells and the phagocytosis of tuberculosis.

## 3 The function of CD36 in *M. tuberculosis* infection

**Figure 2:**
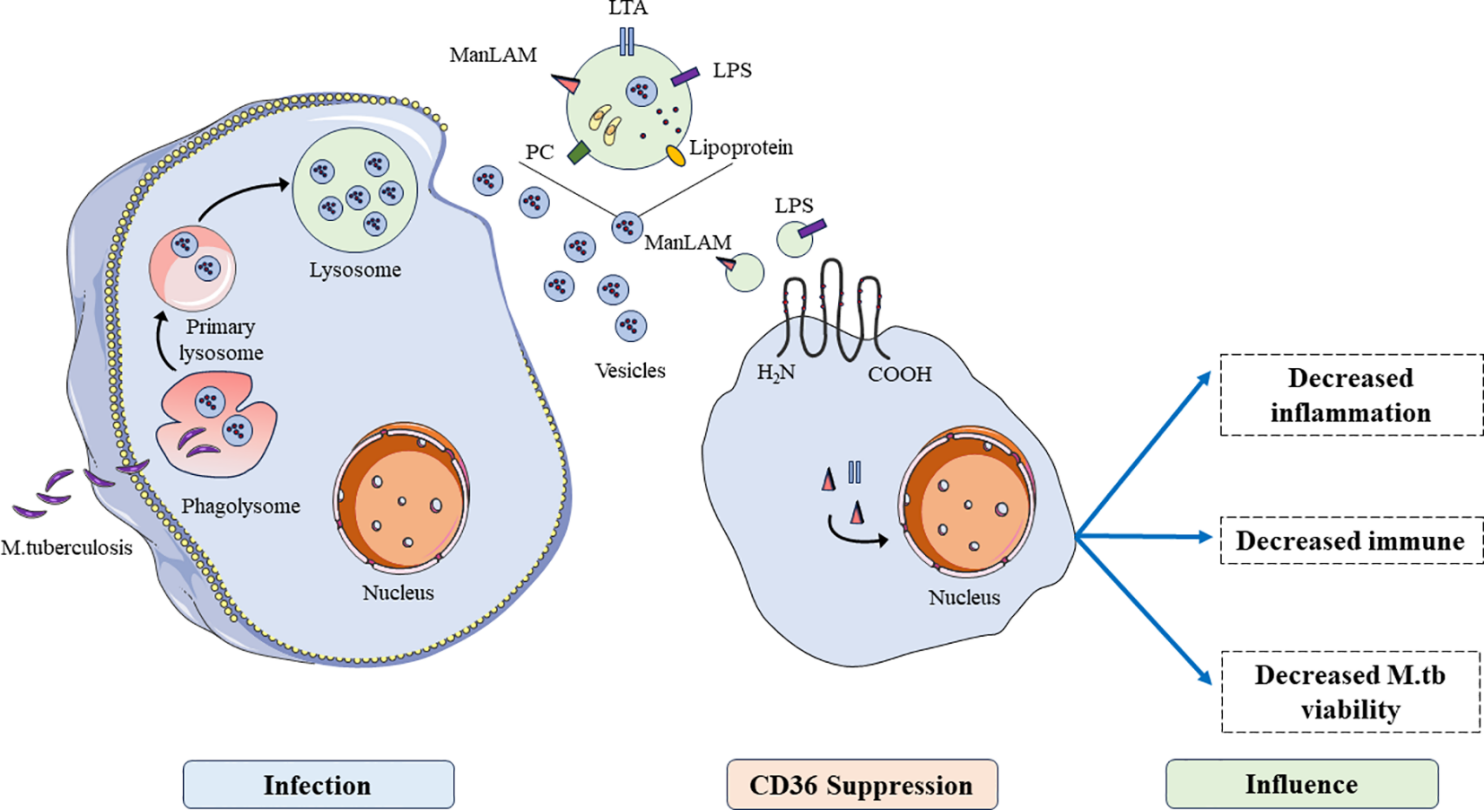
The functions of CD36 in the process of *M. tuberculosis* infection. After being engulfed by host cells and lysosomal lysis, antigens of *M. tuberculosis* are presented to immune cells through vesicles, inhibiting immune cell functions such as inhibiting inflammation, immune function, and promoting viability of *M. tuberculosis*.

After infecting host cells, *M. tuberculosis* generated intracellular phagosome and fused with lysosomes, then the components of *M. tuberculosis* were presented to immune cells through vesicles. Immune cells regulate host cells to produce immunosuppressive responses by uptake of liposomes of *M. tuberculosis* in vesicles through CD36 (**Figure 2**). CD36 has been shown to promote lipid uptake in cells which may serve as a source of carbon to support the cell’s development (68). CD36 acts as a transporter of long chain fatty acid and function its effect on glycolysis and the trichloroacetic acid (TCA) cycle in *M. tuberculosis* infection (69). During this infection, this pathogen promotes the expression of CD36 and make host cells take up more long-chain fatty acids to produced sufficient ATP through glycolysis or the tricarboxylic acid cycle, allowing it to lurk in host cells for a long time and infect surrounding normal cells (70). M1 macrophages require rapid generation of ATP to activate inflammation through glycolysis via the inhibition of OXPHOS and TCA cycle in mitochondria (71, 72). These findings provided a novel target to treat the infection of *M. tuberculosis*.

Pepino et al. found macrophages pretreated with surfactant lipids increased the CD36 expression and induced the translocation of CD36 from the cytosol to the membrane (27). According to Dodd et al., surfactant lipids and/or surfactant protein A (SP-A) can cause preformed CD36 to be redistributed in the cell membrane, which can increase the production of CD36 (68). Advanced glycation end products (AGEs), which are also CD36 ligands, and oxiLDL are produced as a result of oxidative stress (73). The expression of CD36 in cells involved in the inflammation and immune response is dependent on the recognition and the uptake of lipid and metabolic components of *M. tuberculosis* (74, 75). Furthermore, CD36 plays a role in the uptake of surfactant lipids by human macrophages the CD36 knockdown decreases the uptake of dipalmitoylphosphatidylcholine (DPPC) and pre-infection exposure of human macrophages to surfactant lipids promotes *M. tuberculosis* growth in a manner that is CD36-dependent. These findings suggest that CD36 mediates the uptake of surfactant lipids by human macrophages, a function that *M. tuberculosis* exploits for growth. When *M. tuberculosis* is phagocytosed by macrophages, the immune system produces cytokines that target the phagocytes by secreting antimicrobial compounds, attracting PMNs to the infection site and producing granulomas (76). Oxidative stress is produced by granulomas made of immobilized *M. tuberculosis* and infiltrating macrophages to destroy the immobilized bacteria (77).

Hematopoietic stem cells (HSCs) also require CD36-mediated free fatty acid (FFA) uptake to compensate for metabolic requirements during acute infections (78). Once the components or metabolites of *M. tuberculosis* circulate in the peripheral blood, HSCs rapidly produce a large number of monocytes, T lymphocytes, and dendritic cells, promoting the transformation of monocytes into macrophages to cope with *M. tuberculosis* infection (79). Mistry et al. revealed the significant roles of CD36 in HSC metabolism in response to acute infections, which is impaired if CD36-mediated FFA uptake is absent and leads to increased mortality in HSCs (80). Meanwhile, *M. tuberculosis* not only could alter hematopoiesis by directly infecting the bone marrow niche harboring HSC, but also induce systemic cytokines such as TPO, SCF, IL-3, IL-6, TGF-β, MIP-1α and so on during chronic infection (81, 82).

However, monocytes and macrophages are crucial in the formation of granuloma.

Castaño et al. reported that monocytes infected by *M. tuberculosis* produced fewer granules and decreased the number of cytoplasmic projections and the expression of CD36, CD86 and CD68 compared to monocytes differentiated in the absence of mycobacteria (25). Monocytes treated with recombinant IL-1b prevented the increased expression of HLA-II, CD86 and CD36 observed with differentiation into macrophages (25). Moreover, infected monocytes suppressed the secretion of IL-6, TNF-a, IL-10, IL-12p70, but promoted the expression of IL-1b in response to LPS and purified protein *M. tuberculosis*-derived (25). According to a recent work by Baranova et al., CD36 mediates signaling triggered by gram-negative bacteria and LPS via a JNK-mediated signaling pathway in a manner that is TLR2/4-independent and serves as a phagocytic receptor for a range of bacteria (83). Furthermore, Józefowski et al. demonstrated that an antibody-neutralizing scavenger receptor SR-PSOX/CXCL16 partially reversed the augmentation of LPS-induced TNF-a generation by dextran sulphate, whereas an antibody-neutralizing CD36 reversed the stimulatory effect of deacylated ManLAM (84). According to this study, NO generation is regulated by unidentified scavenger receptors, whereas CD36 regulates the activity of ManLAM and its deacylated form that results in TNF-a release in LPS-stimulated J774 cells and peritoneal murine macrophages (84). Accordingly, *M. fortuitum* which was unable to synthesize ManLAM was not bound by murine CD36 expressed in HEK293 cells, indicating that CD36 is required for the uptake of mycobacteria (85). Depending on the route of infection, CD36 may have distinct effects on interactions between mycobacteria and distinct populations of macrophages that mycobacteria first encounter *in vivo*. Alemán et al. found that the phagocytosis of *M. tuberculosis*-induced neutrophils by immature dendritic cells (iDCs) leads to lymphoproliferation, which is significantly reduced by blocking CD36 and not DC-SIGN on iDCs (86). However, Hedlund et al. revealed that dendritic cells (DC) from *M. tuberculosis*-induced apoptotic neutrophils contained almost all stimulatory capacity, and the cell contact-dependent activation required binding of CD11b/CD18 to the DC via DC-SIGN but did not involve CD36 indicating that the cell interaction is crucial for DC activation (87). Of course, these studies reflected differences in CD36 expression and other *M. tuberculosis* sensing receptors, determining macrophage responses to *M. tuberculosis*.

CD36 is also a lysosomal membrane protein and plays critical roles in lysosomal enzyme trafficking and uptake of pathogens, respectively and generally in host cell defences against intracellular pathogens (88). LmpB is a functional homologue of CD36 and could specifically mediate the uptake of mycobacteria and lysosome biogenesis (89). This evidence indicated the functions of LmpA and LmpB as ancestors of the family of LIMP-2 and CD36, in lysosome biogenesis and host cell defence (89). Almeida et al. revealed that the synergistic interactions between CD36 and TLR2 are responsible for the altered lipid metabolism of host cells caused by infection. This data showed that CD36-TLR2 cooperation and signaling compartmentalization inside rafts, via PPAR-γ dependent and NF-κb independent pathways, reroute host response signaling, thereby promoting lipid accumulation and down-regulating the response of BCG-infected macrophages (90) (80). BCG induced CD36 expression and CD36’s roles were further validated by the inhibition of BCG-induced lipid body formation *in vitro* and *in vivo* in macrophages from CD36 deficient animals. Hawkes et al. showed CD36-/- mice showed decreased levels of inflammatory factors, the density of granuloma and the burden of *M. tuberculosis* in the spleen and liver during *M. tuberculosis* infection (91). The viable bacteria in macrophages from CD36-/- mice and the growth of *M. tuberculosis* were decreased significantly (91). These results fully demonstrated that CD36 regulated cellular lipid uptake and metabolism through various signaling pathways containing TLR receptors, PPAR-γ and NF-κb pathways, as well as eliminating intracellular mycobacteria through lysosomes and endocytosis.

**Figure 3:**
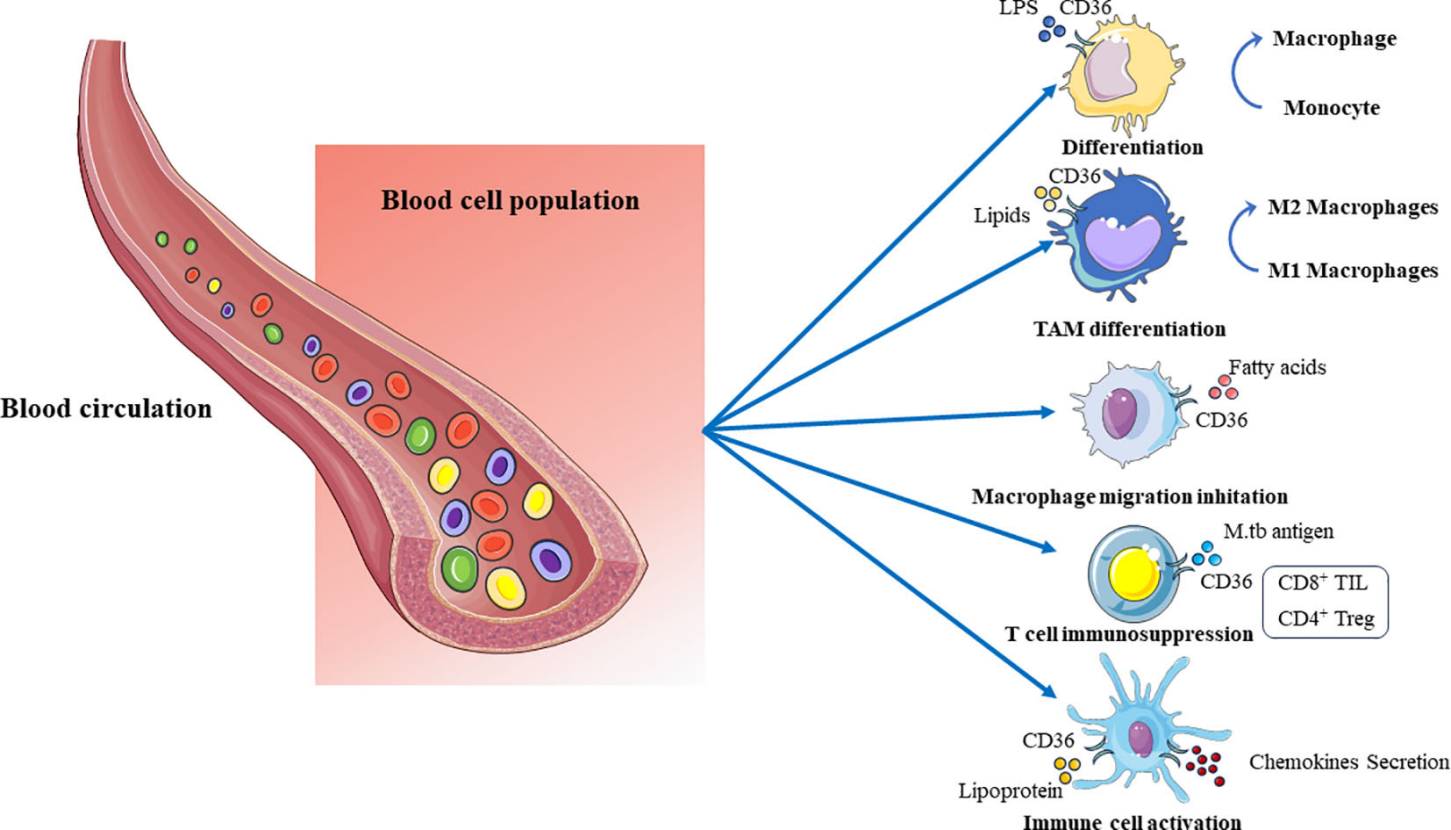
The lipid regulatory function of CD36 on immune cells in peripheral blood cells. CD36 promotes the transformation of mononuclear cells into macrophages during lipid metabolism, M2 polarization of macrophages, inhibition of macrophage migration, promotion of immune cell activation and secretion of cytokines, inhibition of T cell activation, and other functions.

In summary, after infection with *M. tuberculosis*, CD36 protein could induce the transformation of mononuclear cell into macrophages, polarization of M2 macrophages, promotion of cytokine secretion, inhibition of macrophage migration and T cell activation, resulting in inflammatory response and specific cellular immune suppression (**Figure 3**). Preliminary studies at the cellular and animal model levels have shown the functional role of CD36 in the immune response to tuberculosis infection, but the specific molecular mechanisms still need to be further investigated.

## 4 The potential function of CD36 in the treatment of tuberculosis

Based on the diverse immune regulatory functions of CD36 in *M. tuberculosis* infection and its ability to regulate pathogen growth, CD36 has become a potential key target for tuberculosis treatment. Current research on the roles of CD36 in mycobacterial infections has demonstrated the intricate nature of interactions between macrophages and *M. tuberculosis*. These studies used genetically deficient mice to study the functions of CD36 in anti-mycobacterial host defense, with qualitatively different findings (91, 92). Court et al. reported that the long-term regulation of *M. tuberculosis* infection is unaffected by the absence of CD36 plus SR-A (92). After being challenged with *M. bovis BCG*, animals lacking CD36 alone had lower mycobacterial loads and granulomatous reactions, as demonstrated by Hawkes et al. (91). These findings indicated that CD36 is a receptor that mycobacteria use to infiltrate macrophages and demonstrated CD36’s role in cellular scenarios related to granuloma formation, which facilitate early bacterial growth and spread. This evidence indicates studies of CD36 in regulating the survival and growth of intracellular mycobacteria are insufficient and controversial which needs further research and suggests that CD36 may be an important valuable target for controlling or eliminating *M. tuberculosis*.

The hallmark of *M. tuberculosis* infection, foam cells, are reduced when stem bromelain-induced macrophages (SBM) cleaves CD36, according to research by Mahajan et al. (93). This creates an environment that facilitates the increased clearance of *M. tuberculosis* and provides a mechanism for the anti-mycobacterial activity of SBM. ManLAM might be the main CD36 ligand on the surface of *M. tuberculosis*. Anti-CD36 mAb, but not anti-TLR2 mAb, counteracted the increasing effect of deacylated ManLAM on the LPS-stimulated TNF-a generation, as reported by Baranova et al. (83). This suggests that CD36 can independently initiate intracellular signaling. In macrophages from immunologically naï¨ve guinea pigs infected with *M. tuberculosis*, Palanisamy et al. demonstrated an increase in CD36 and LOX1, which enhances the uptake of oxidized host macromolecules, including OxLDL (94). The intracellular buildup of OxLDL, the expression of CD36 and LOX1 in macrophages, and the bacterial load were all reduced in guinea pigs immunized with BCG prior to aerosol challenge (94). This study demonstrated that intracellular bacilli survival and persistence were supported by oxidative stress in guinea pigs infected with *M. tuberculosis* and the possible roles of OxLDL-loaded macrophages. This literature has presented various potential treatments or control strategies for *M. tuberculosis* infection through CD36 targets including natural compounds, ligands, etc. More importantly, CD36 may play an important role in the immune process of conjugated vaccines, providing a new perspective for subsequent research.

*M. tuberculosis* infection triggers an immune response that converges on formation of a granuloma, a dynamic and spatially organized tissue structure composed of macrophages, granulocytes, lymphocytes and fibroblasts, and provide the immune microenvironment for *M. tuberculosis* infection (95). In *M. tuberculosis* infected microenvironment, additional phagocytes were recruited to the site of infection through the secretion of cytokines and chemokines from above immune cells (96). In addition, the expression of CD36 is regulated at both the transcriptional and posttranslational levels among these different cells. In monocytes, CD36 is upregulated by PPAR-ɣ, as well as by cytokines including CSF, IL-4, and IL-10 that are involved in differentiation toward DCs and reparative “M2”-like phenotypes (97, 98). These findings provide us with new ideas for treating tuberculosis infection. If the expression of CD36 on various immune cells in granulation was inhibited, the energy metabolism of infected cells will be reduced to decrease the latent proliferation of *M. tuberculosis*. We believe that achieving the goal of treating tuberculosis will not be far away.

## 5 The functions of CD36 as a biomarker for the diagnosis of tuberculosis

CD36, as an important signaling molecule on the cell surface, is expressed on various cell surfaces, and CD36 has become a diagnostic biomarker for various tumors (99, 100). CD36 also has important potential value in the diagnosis of tuberculosis infection. *In vitro*, Sánchez et al. also found that *M. tuberculosis* infection decreased the expression of CD36 correlated with induction of apoptosis indicating that the low expression of CD36 is closely related to tuberculosis infection (101). Shkurupy et al. found CD36, CD11 and CD29 on macrophages increased significantly due to *M. tuberculosis* infection and participated in macrophages fusion suggesting that the expression of these molecules is a constitutive property of peritoneal (102). CD36 was up-regulated by *M. tuberculosis* H37Ra infection in macrophages and suppressed in exosomes from H37Ra-infected macrophages indicating that CD36 could be a potential biomarker associated with TB infection (103).

**Figure 4:**
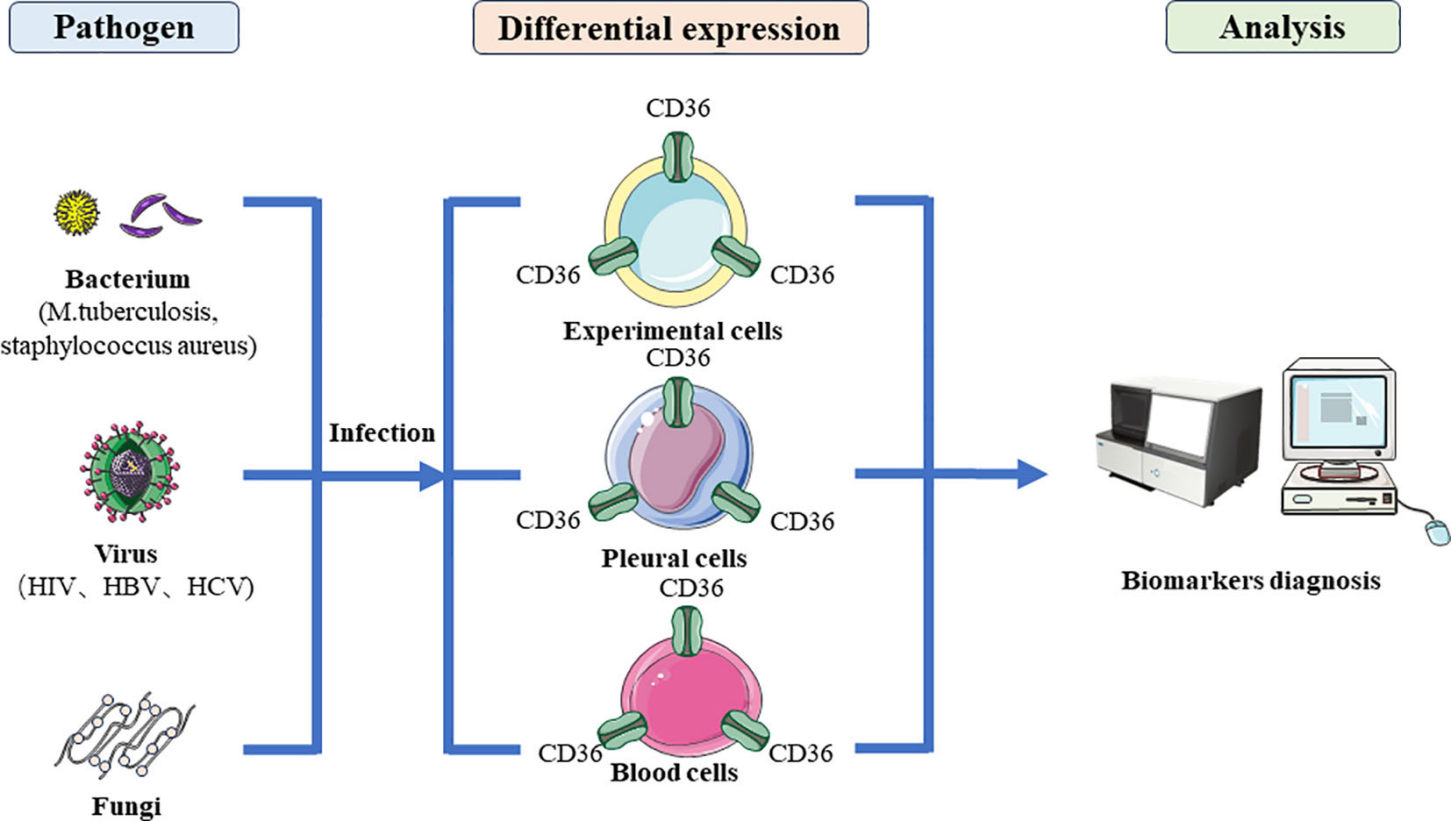
The diagnostic application of CD36 in microbial infections. After bacterial, viral and fungal infection, they regulate the specific expression of CD36 protein in host cells, making CD36 a marker for diagnosing microbial infection.

Currently, *in vivo*, numerous samples of evidence indicate that CD36 also demonstrates its potential as a diagnostic biomarker (**Figure 4**). Single nucleotide polymorphisms (SNP) in CD36 indicated the risk of pulmonary tuberculosis is decreased in SNPs due to the reduced ability of CD36 to recognize the *M. tuberculosis* pattern recognition molecules indicated CD36 as important receptors in response to PTB (104). There is evidence indicating that TGF-E down-regulated CD36 expression and the upexpression of TGF-E existing may be responsible for the downexpression and decreased percentage of CD36+ monocytes in TB patients (105, 106). The reduction in CD36+ monocytes suggests that TB patients’ monocytes and macrophages would be less able to identify and eliminate apoptotic cells which would cause the cells to become late necrotic and promote the spread of germs (106). In patients with various clinical manifestations of tuberculosis, Sánchez et al. had previously shown an increased frequency of CD14+ monocytes along with decreased expression of CD36 and HLA-DR, indicating that immature cells were circulating during active TB (107). Flow cytometry was used to evaluate blood mononuclear cells from TB patients with varying clinical phases for the markers CD36, CD14, CD40, CD163 and CD203. However, CD36 and CD14 were found to be reduced in TB patients (107). Zhang et al. found serum exosomal protein CD36 decreased significantly in active TB (ATB) patients, and CD36 was downexpressed in peripheral blood mononuclear cells (PBMCs) of ATB patients through the comprehensive proteomics analysis (108). Similarly, among LTBI individuals with coronary artery disease (CAD), CD36 was down expressed across all monocyte subsets suggesting that the lower expression of CD36 could engender a negative feedback mechanism to counterbalance ongoing inflammation (109).

A large amount of clinical trial data indicated that CD36 is downregulated in the peripheral blood of tuberculosis patients, which is beyond doubt. The abnormal expression of CD36 is only analyzed at the cellular level and peripheral blood *in vivo*, and it is still far from becoming a diagnostic kit for clinical application. At least the following issues need to be addressed: (1) The abnormal expression of CD36 is specific to *M. tuberculosis* infection and is not affected by other diseases; (2) The sensitivity and standards of CD36 for detecting *M. tuberculosis* infection need to be studied; (3) The commercialized CD36 reagent kit needs further optimization. Despite facing numerous challenges, researches on the correlation between CD36 and *M. tuberculosis* infection have laid an experimental and theoretical foundation for CD36 to become a diagnostic marker of tuberculosis.

## 6 Future prospects

At present, the scavenger receptor CD36 plays an important role in tumors and lipid metabolism and is a critical hotspot in these fields. However, in recent years, researchers found that CD36 also acted as a significant influence on the recognition of ligands from pathogens especially in *M. tuberculosis* and host cells. CD36 mainly involves the lipid metabolism, inflammatory response, immune response, vaccine immunity, and diagnostic markers of hosts infected with *M. tuberculosis*. The functions of CD36 in *M. tuberculosis* infection has gradually become clear, and numerous studies are conducting animal and drug experiments targeting CD36, hoping to therapy tuberculosis. However, there have been few significant achievements so far, and clinical trials are far from being conducted. At the same time, the clinical diagnosis of tuberculosis infection by CD36 is still in the initial exploration stage both *in vivo* and *in vitro*, and requires a large amount of clinical testing to confirm its diagnostic value.

This study comprehensively reviews the relevant roles and functions of CD36 in tuberculosis infection, providing a research foundation for researchers. Although CD36 has a wide range of functions in the process of *M. tuberculosis* infection in hosts, related studies are still inadequate, and the specific molecular mechanisms in related fields are still not clear enough.

## Author contributions

JW: Conceptualization, Data curation, Funding acquisition, Investigation, Writing – original draft, Writing – review & editing. HC: Investigation, Resources, Supervision, Writing – original draft. HY: Data curation, Formal analysis, Writing – original draft. NW: Methodology, Software, Visualization, Writing – review & editing. YW: Formal analysis, Validation, Writing – review & editing. HL: Formal analysis, Project administration, Writing – review & editing.
